# Microbial Community Structure in a Malaysian Tropical Peat Swamp Forest: The Influence of Tree Species and Depth

**DOI:** 10.3389/fmicb.2018.02859

**Published:** 2018-12-04

**Authors:** Chin Chin Too, Alexander Keller, Wiebke Sickel, Sui Mae Lee, Catherine M. Yule

**Affiliations:** ^1^School of Science, Monash University Malaysia, Subang Jaya, Malaysia; ^2^Department of Animal Ecology and Tropical Biology, Biocenter, University of Würzburg, Würzburg, Germany; ^3^Center for Computational and Theoretical Biology, University of Würzburg, Würzburg, Germany; ^4^Tropical Medicine & Biology Multidisciplinary Platform, Monash University Malaysia, Subang Jaya, Malaysia; ^5^School of Science and Engineering, University of the Sunshine Coast, Sippy Downs, QLD, Australia

**Keywords:** tropical peat swamp forest, metabarcoding, microbial diversity and composition, tree species, depth, methanogens

## Abstract

Tropical peat swamp forests sequester globally significant stores of carbon in deep layers of waterlogged, anoxic, acidic and nutrient-depleted peat. The roles of microbes in supporting these forests through the formation of peat, carbon sequestration and nutrient cycling are virtually unknown. This study investigated physicochemical peat properties and microbial diversity between three dominant tree species: *Shorea uliginosa* (Dipterocarpaceae), *Koompassia malaccensis* (legumes associated with nitrogen-fixing bacteria), *Eleiodoxa conferta* (palm) and depths (surface, 45 and 90 cm) using microbial 16S rRNA gene amplicon sequencing. Water pH, oxygen, nitrogen, phosphorus, total phenolic contents and C/N ratio differed significantly between depths, but not tree species. Depth also strongly influenced microbial diversity and composition, while both depth and tree species exhibited significant impact on the archaeal communities. Microbial diversity was highest at the surface, where fresh leaf litter accumulates, and nutrient supply is guaranteed. Nitrogen was the core parameter correlating to microbial communities, but the interactive effects from various environmental variables displayed significant correlation to relative abundance of major microbial groups. Proteobacteria was the dominant phylum and the most abundant genus, *Rhodoplanes*, might be involved in nitrogen fixation. The most abundant methanogens and methanotrophs affiliated, respectively, to families Methanomassiliicoccaceae and Methylocystaceae. Our results demonstrated diverse microbial communities and provide valuable insights on microbial ecology in these extreme ecosystems.

## Introduction

Tropical peat swamp forests (TPSF) are among the most exploited but least scientifically studied ecosystems in the world. They are found extensively in Southeast Asia (about 56% of total tropical peatlands in the world), particularly in Indonesia (∼47%) and Malaysia (∼6%) (Page et al., 2011). Most TPSF are ombrogenous, where nutrient inputs depend solely on the rainfall, marine aerosols and leaf litter (Ong et al., 2015). Peat accumulates due to the inhibition of the microbial activities caused by waterlogged anaerobic conditions and the recalcitrant plant detritus, resulting in an acidic, toxic and phenol-rich peat substrate (Yule et al., 2016). In comparison with the northern peatlands that are covered in mosses and sedges, TPSF support highly biodiverse forests. For instance, Anderson (1963) recorded 927 plant species in the TPSF of Borneo, and there were 260 plant species documented in Pekan Peat Swamp Forest in Peninsular Malaysia (Latiff, 2005). The substrates of tropical and northern peatlands share similarities with respect to waterlogging, acidity and low levels of nutrients, but the origins of the peat differ – in the tropics, they are derived from plant debris such as leaves, roots, trunks and branches compared with the mosses, grasses, sedges and shrubs of northern peatlands. Tropical peat formation has typically been occurring over thousands of years since the last glaciation. Its annual accumulation rate is estimated at 2–5 mm, which is much faster than in boreal peatlands due to high productivity, and there have been reports of peat layers up to 20 m thick (Yule, 2010).

TPSF play a prominent role in the global carbon cycle as enormous carbon stock reservoirs, storing an estimate of 89,000 teragrams (1 Tg = 1 billion kg) of organic carbon (Moore et al., 2013). Leete (2006) estimated that a 10-m deep peat swamp stores about 5,800 tons of carbon per hectare compared to about 300–500 tons per hectare for other types of tropical forests. Most SE Asian TPSF have been degraded or destroyed over recent decades by logging, drainage and fires (Miettinen et al., 2011). Such degradation triggers aerobic decomposition of the peat and leads to significant greenhouse gas (e.g., CO_2_, CH_4_, and N_2_O) emissions that exacerbate global warming. Therefore, conservation and rehabilitation on TPSF is urgently needed for effective mitigation of global climate.

Studies conducted on TPSF thus far have mostly focused on the issues of carbon storage and greenhouse gasses emissions (Page et al., 2002; Inubushi et al., 2003, 2005; Jauhiainen et al., 2005; Melling et al., 2005; Toma et al., 2011; Turetsky et al., 2015), hydrology (Wösten et al., 2008a), leaf litter decomposition (Yule and Gomez, 2008; Ong et al., 2015), restoration and conservation (Phillips, 1998; Wösten et al., 2008b; Page et al., 2009; Yule, 2010). There is still a paucity of information regarding the microbial ecology of TPSF. Microbes are key drivers in various biogeochemical processes, such as carbon and nitrogen cycles, for efficient function and sustainability of the ecosystems. Therefore, questions arise, how do TPSF support forests of such high biomass and diversity on a substrate of peat? What are the roles of microbes in supporting these forests through peat formation, nutrient cycling and carbon sequestration? It is therefore essential to study the microbial community dynamics in order to understand the functioning of such an extreme ecosystem. Understanding the microbial ecology of TPSF will greatly assist in their management and conservation, to maintain peat accretion, protect their carbon sequestration capacity and to assist in the rehabilitation of peatlands degraded by clearing, drainage, fires and agricultural conversion.

There have been few papers documenting microbial diversity in TPSF in Malaysia (Jackson et al., 2009), Thailand (Kanokratana et al., 2011), and Brunei (Tripathi et al., 2016). Their results demonstrated that both Proteobacteria and Acidobacteria are among the most dominant phyla in TPSF. There were no methanogens detected in the Malaysian TPSF (Jackson et al., 2009) but methanogens from the class Methanomicrobiales were found to be abundant in the Thai TPSF (Kanokratana et al., 2011). In addition, there have been several studies conducted on specific bacterial strains in TPSF. For example, the discovery of antimicrobial properties in novel bacterial strains isolated from TPSF in Peninsular Malaysia by Aw et al. (2016) and Ong et al. (2016), as well as the isolation of two *Burkholderia* strains also from Peninsular Malaysian TPSF with the ability of lignin degradation (Roslan et al., 2015). Arai et al. (2014) studied the impact of drainage and fire on the methanotrophic bacterial community of a peatland in Indonesia and concluded that *Methylomonas* spp. were unaffected by such disturbance.

This study investigated microbial community structure (diversity, composition and relative abundance) between tree species and depths in NSPSF, taking into account the impacts of environmental factors (water and peat pH, dissolved oxygen, organic carbon content, total nitrogen, total phosphorus, total phenolic content and C/N ratio) on microbial communities. Three common tree species in NSPSF were selected: *Shorea uliginosa* (family Dipterocarpaceae), *Koompassia malaccensis* (legumes in the family Fabaceae that is associated with nitrogen-fixing bacteria) and *Eleiodoxa conferta* (family Arecaceae). *Shorea* and *Koompassia* are emergent trees that possess distinct physicochemical properties and apply different strategies for nutrient cycling (Ong et al., 2015). *Eleiodoxa* is a colony-forming palm tree that requires wet conditions. These species were selected as they were among the most abundant tree species in TPSF, and thus they are expected to contribute a relatively large amount of plant debris and have a relatively important influence on peat formation. Moreover, *Shorea* and *Koompassia* were studied previously (Ong et al., 2015), so aspects of their ecology and nutrient dynamics with respect to leaf decomposition are known and facilitated this study.

The following hypotheses were tested:

(i) *Koompassia* is expected to possess the highest microbial diversity compared to two other tree species, as its association with nitrogen-fixing bacteria enables it to receive nitrogen supply directly at the roots for microbial metabolism.
(ii) The surface, where fresh leaf litter accumulates and provides nutrients via leaching, is expected to show the highest microbial diversity.
(iii) Methanomicrobiales are expected to be the most abundant methanogens found in the ombrotrophic TPSF, with increasing abundance in the deeper old peat due to the anaerobiosis in deeper layers.
(iv) Type II methanotrophs, the family Methylocystaceae, are expected to be found as the most prevalent methanotrophs, with the highest abundance at the surface to guarantee sufficient oxygen supply for methane oxidation.

## Materials and Methods

### Study Site

**FIGURE 1:**
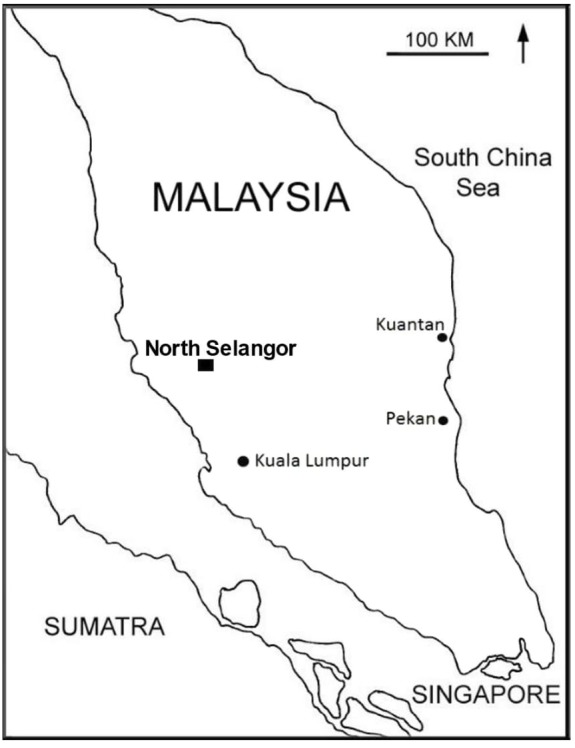
Location of North Selangor peat swamp forest on the map of Peninsular Malaysia.

NSPSF is located on a flat coastal plain in NW Selangor, Malaysia (N 3°39′08.3^′′^, E 101°17′58.1^′′^, ∼5 m above sea level) (Figure 1). The forest covers 81,304 ha and comprises four forest reserves. It experiences a tropical climate of heavy rainfall with high temperature and humidity. The mean monthly rainfall is 136–248 mm, typically with peaks in March–April and October–November. Dry seasons usually fall between May–September. The average temperature is 27°C and the mean relative humidity is 79.3% (GEC, 2014). The deepest peat is recorded at 10.15 m (GEC, 2014). NSPSF is estimated to contain 132 million tons of carbon, which is equivalent to 473 million tons of carbon dioxide (GEC, 2014). NSPSF receives water inflow mainly from the rainfall, and occasionally overflow from Bernam River. Over 324 species of trees, mosses, orchids and herbs have been recorded in NSPSF including emergent trees over 30 m, such as *K. malaccensis, S. uliginosa, Xylopia fusca, Santiria* sp., and *Syzygium* sp (GEC, 2014).

### Sample Collection

Sample collection was conducted on 5 Dec 2014, which was during the wet season, and thus all sampling sites were submerged. Sampling sites were selected haphazardly based on the presence of tree species of interest. Individuals of the same tree species were at least 100 m apart, but the individuals of different tree species were sometimes close to each other (≥10 m). Three individual trees of each species were chosen, and peat samples were collected from three sites around the rhizosphere of each tree, within 2 m from the trunk of the tree (*Shorea* and *Koompassia* have large buttress roots and thus it was not possible to have a standard distance from the trunk). For each site, samples were collected at three depths: surface, 45 and 90 cm using a soil corer. Rubber gloves and sterile spatulas were used for sampling and the samples were kept in sterile micro-centrifuge tubes. A total of 81 samples for molecular analyses were collected. The samples were stored at -20°C until further analysis in the laboratory.

In addition, peat samples from each depth were also collected within 2 m from the trunk of each individual tree for environmental analyses: peat pH, organic carbon content, total nitrogen, total phosphorus, total phenolic content and C/N ratio. A total of 27 peat samples were collected and placed in ziplock bags. They were stored at 4°C until further analysis in the laboratory. Water temperature, pH and dissolved oxygen at each depth for every individual tree were measured *in situ* using a multimeter (Eutech Instrument Multi meter – PD 650).

### Environmental Characteristics Measurements and Analyses

#### Peat pH

Peat samples were oven-dried at 60°C and sieved through 1-mm mesh (Yesmin et al., 1996). MiliQ water was added to the sieved peat at a ratio of 1:5 and the pH was taken using a pH meter (Eutech Instrument Multi meter – PD 650). The process was repeated using 0.25 M calcium chloride solution (CaCl_2_), as it provides a more stable pH in peat (Parent and Tremblay, 2002).

#### Organic Carbon Content

The organic carbon in the peat is released as CO_2_ during combustion and thus ash-free dry mass (AFDM) indicates the organic carbon content of the peat (Clinton et al., 2010). About 3–5 g of peat was weighed and dried in an oven at 60°C for 3 days. The dried peat was then combusted in a furnace at 550°C for 6 h and the ash was weighted. The calculation was done following Agus et al. (2011).

$$\begin{array}{l} \text{AFDM=Mass of dried peat - mass of ash}\\ \text{Organic carbon content=[(AFDM/mass of dried peat)}\times \text{100\%]/1.724} \end{array}$$

#### Total Nitrogen (TN) and Total Phosphorus (TP)

Total nitrogen of the samples was determined using the Kjeldahl method (Bremner et al., 1996), and TP was determined based on the protocol of Jones (2001) and Ong et al. (2015). The analyses were conducted by the Soil Chemistry Laboratory in the Faculty of Agriculture, Universiti Putra Malaysia (UPM).

#### Total Phenolic Content (TPC)

Total phenolic content was determined using Folin–Ciocalteu assay (Kähkönen et al., 1999). The phenol compounds were extracted using 70% acetone at 4°C for 12 h. The supernatant was obtained and underwent dilutions in the cases of excessive phenolic content. The supernatant was then added with Folin–Ciocalteu at the ratio of 1:10 and 7.5% sodium carbonate. The absorbance readings were taken at 760 nm on a spectrophotometer (Tecan, Crailsheim, Germany). A standard curve was plotted with tannin acid of 10, 20, 40, 60, 80, and 100 ppm.

#### C/N Ratio

C/N ratio refers to the ratio of organic carbon content to total nitrogen of a substrate measured in mass. It is a common indicator of the degree of decomposition based on the litter quality (Taylor et al., 1989). The values can be calculated from the dataset of the organic carbon content and total nitrogen.

#### Statistical Analyses

All environmental data were tested for normality using the Shapiro-Wilk test, and homogeneity of variance using Levene’s test. In order to examine the significant differences in the environmental data between tree species and depths, two-way analysis of variance (ANOVA) with *post hoc* Tukey HSD test were applied for the normally distributed data. For non-normally distributed environmental data, the Friedman Test was performed along with Wilcoxon signed-rank test as *post hoc*, which was conducted with a Bonferroni correction. In the cases where the environmental characteristics displayed significant differences only between depths, all data were pooled to the depths and one-way ANOVA was implemented along with the *post hoc* Tukey HSD test for normally distributed data, while Kruskal-Wallis test with Mann-Whitney as *post hoc* for non-parametric data. All statistical analyses were conducted in SPSS v16.0 (SPSS, Inc., Chicago, IL, United States), and the significance value was set at *p* < 0.05 throughout the study, unless stated otherwise.

### Metabarcoding Protocols and Analyses

#### DNA Extraction

DNA extraction was conducted directly from peat samples using MoBio PowerSoil DNA Isolation Kit (MoBio Laboratories, Carlsbad, CA, United States) according to the manufacturer’s protocol (Jackson et al., 2009). The isolated DNA were stored at -20°C until further molecular analysis.

#### Library Preparation and 16S rRNA Gene Amplicon Sequencing

DNA amplification targeted at the V4 region of 16S rRNA gene was conducted with a dual-indexing strategy according to Kozich et al. (2013). Adapters and dual-indices were incorporated directly into the PCR primers to allow multiplexing of the samples. PCR products were then purified and normalized in DNA amounts using the SequalPrep Normalization Plate Kit (Invitrogen) and quality checked on a Bioanalyzer using a High Sensitivity DNA kit (both Agilent Technologies, Santa Clara, CA, United States), followed by sequencing using MiSeq Reagents kit v2 of 2 × 250 bp output on the Illumina MiSeq platform in Biocenter, University of Würzburg. Illumina PhiX control kit v3 was added with 5% of total DNA amount to account for low sequence diversity.

#### Sequencing Data Processing

Read quality was evaluated using *FastQC* v0.11.2 (Andrews, 2010). Data analysis followed Junker and Keller (2015), in which a Linux shell script of used software and parameters was also provided as Supplementary Material and used as a basis for analysis here. Major steps were: In *QIIME* v1.8.0 (Caporaso et al., 2010), forward and reverse reads were joined with fastq-join v1.8.0, and reads were quality filtered at Q20. Operational taxonomic units (OTUs) were clustered and chimeras were removed using the *USEARCH* (Edgar, 2010) and *UCHIME* algorithms (Edgar et al., 2011) in *USEARCH* v7.0.1090 (Edgar, 2010). OTUs were then taxonomically assigned based on the *Greengenes* reference database (DeSantis et al., 2006), and converted into the BIOM format in *QIIME*. OTUs belonging to chloroplast, chlorophyte, mitochondrial and those unassigned at kingdom level were filtered. RAxML was used to construct a phylogenetic tree using all remaining OTUs (Stamatakis et al., 2008).

#### Sequencing Data Analysis

The BIOM file was imported to *R* v3.2.2 for the downstream analysis. For alpha diversity, the OTU richness, Shannon diversity index (SDI) and Faith’s phylogenetic diversity (PD) were calculated using the *R* package *phyloseq* (McMurdie and Holmes, 2012). Further, non-metric multidimensional scaling (NMDS) with 999 permutations were performed based on Bray-Curtis dissimilarity and weighted *UniFrac*. Bray-Curtis dissimilarity quantifies the taxa abundances dissimilarity across samples, whereas weighted *UniFrac* distances is a distance metric that incorporates the phylogenetic distance of the taxa across the samples. A heatmap showing the distribution of phyla along the depth profile was created in *ggplot2* (Wickham, 2016). The permutational ANOVA test with 999 permutations was conducted in *vegan* (Oksanen et al., 2007) to compare microbial communities between each factor (Anderson, 2001). In addition, environmental fitting (*envfit*) of the *vegan* package, which determines the significance by random permutations, was applied to identify the correlation between environmental characteristics and the microbial communities. For examining the correlation between various environmental characteristics with the relative abundance of OTUs in various microbial phyla, classes and orders, Pearson’s correlation coefficient was employed for normally distributed data or Spearman’s rank correlation coefficient for non-normally distributed data in SPSS v16.0. The significance level was set at *p* < 0.05 for all statistical tests.

#### Nucleotide Sequence Accession Numbers

The nucleotide data associated with this study is publicly accessible at the European Nucleotide Archive^1^ under accession number PRJEB22141.

## Results

### Environmental Characteristics

Environmental characteristics of the studied tree species and depths are listed in Supplementary Table S1. As tree species did not show significant impact on any environmental characteristics, all measurements were pooled according to the depth for downstream analyses. Environmental characteristics that were found significantly different between depths are shown in Table 1.

**Table 1:** Environmental characteristics based on the depth in NSPSF.

Depth	
(cm)	Water pH	DO (mg/l)	TN (%)	TP (μg/g)	TPC (mg TAE/g)	C/N ratio
0	3.49 ± 0.03^a^	1.64 ± 0.02^a^	1.97 ± 0.08^a^	233.09 ± 6.89^a^	105.38 ± 14.50^a^	28.65 ± 1.22^a^
45	3.35 ± 0.07^ab^	0.95 ± 0.07^b^	1.63 ± 0.05^a^	142.18 ± 7.76^b^	208.31 ± 13.61^b^	34.13 ± 1.21^a^
90	3.17 ± 0.07^b^	0.64 ± 0.06^c^	1.20 ± 0.10^b^	74.15 ± 14.99^c^	248.90 ± 24.35^b^	49.99 ± 4.84^b^

*Different letters indicate significant differences between depths at *p* < 0.05. All values shown are mean ± SE. DO, dissolved oxygen; TN, total nitrogen; TP, total phosphorus; TPC, total phenolic content*.

Water temperature was stable across all sampling sites, ranging between 25.2 and 26.5°C. Depth significantly influenced water pH [χ^2^ (2) = 8.667, *p* = 0.013], DO [*χ*^2^ (2) = 18.000, *p* < 0.001], TN [*F*_(2,18)_ = 14.997, *p* < 0.001], TP [*χ*^2^ (2) = 18.000, *p* < 0.001], TPC [*F*_(2,18)_ = 13.918, *p* < 0.001], and C/N ratio [*F*_(2,18)_ = 11.659, *p* = 0.001]. Deeper peat layers displayed significantly lower water pH, DO, TN, TP but higher TPC and C/N ratio (Table 1). Both peat pH and organic carbon content were not affected by depth (*p* > 0.05).

### Metabarcoding

Sequencing of 81 samples yielded a total of 4,961,719 raw reads and after quality and chimera filtering, there were 3,620,842 reads. Further removal of chloroplast, Chlorophyta and mitochondrial genes left a total of 1,469,860 reads for downstream analysis. Eighteen samples that yielded less than 1,000 reads, respectively, were excluded from the downstream analysis to avoid potential bias due to small sample size. There were 41% of OTUs that could not be taxonomically classified at the genus level based on a cut-off value of 97% using the sequences in GenBank (Supplementary Table S2), indicating TPSF as a reservoir of potentially novel bacterial species. There were in total 5,830 microbial taxa included in the analysis, including 53 archaeal taxa.

The rarefaction curve did not reach plateau (Supplementary Figure S1A), indicating insufficient sequencing effort to represent the entire microbial communities in NSPSF. This is relatively challenging to be achieved for soil samples, as soils usually possess extremely diverse microbial communities (Daniel, 2005). However, when the taxa filtration was applied at a threshold of 0.01% among the total number of reads in each sample, the rarefaction curves were saturated (Supplementary Figure S1B). This suggested that only taxa of very low abundance were missing from the sequencing data.

### Microbial Alpha Diversity

Microbial alpha diversity was assessed by the OTU richness, Shannon diversity index (SDI) and Faith’s phylogenetic index (PD). The number of OTUs in all samples ranged from 451 to 1965, with the highest richness found at the surface and the lowest at 45 cm (Table 2A). Depth contributed significant impact on OTU richness (*F*_(2,60)_ = 4.235, *p* = 0.019), SDI (*F*_(2,60)_ = 7.537, *p* = 0.001) and Faith’s PD (*F*_(2,60)_ = 3.716, *p* = 0.030). Samples from the surface displayed significantly higher OTU richness (*p* = 0.018), SDI (*p* = 0.003), and Faith’s PD (*p* = 0.023) compared to samples at 45 cm. Samples at 90 cm only displayed significantly lower SDI than surface samples (*p* = 0.010). Overall, the highest alpha diversity was observed at the surface, followed by at 90 cm and the lowest at 45 cm (Table 2A). Microbial alpha diversity did not differ significantly between tree species in all measurements (*p* > 0.05).

**Table 2:** Alpha diversity measured in OTU richness, Shannon diversity index and Faith’s phylogenetic diversity (PD) (A) between depths for the overall microbial communities and (B) between depth and tree species for only the archaeal communities in NSPSF.

Depth (cm)	OTU richness		Shannon diversity index	Faith’s PD
**(A)**				
0	1412.85 ± 57.47^a^		5.56 ± 0.09^a^	155.88 ± 5.51^a^
45	1097.17 ± 91.91^b^		5.08 ± 0.11^b^	128.68 ± 8.06^b^
90	1207.42 ± 98.58^ab^		5.15 ± 0.11^b^	142.53 ± 8.45^ab^

		**OTU richness**	**Shannon diversity index**	**Faith’s PD**

**(B)**				
Depth (cm)	0	18.69 ± 1.24^a^	2.07 ± 0.07	9.83 ± 0.48^a^
	45	22.61 ± 1.85^ab^	1.91 ± 0.07	9.88 ± 0.69^a^
	90	26.63 ± 1.75^b^	1.88 ± 0.07	11.55 ± 0.20^b^
Tree species	E	20.31 ± 2.12	1.82 ± 0.11^a^	10.07 ± 0.68
	K	22.35 ± 1.82	1.90 ± 0.06^ab^	10.39 ± 0.49
	S	23.33 ± 1.30	2.13 ± 0.04^b^	10.53 ± 0.45

*All values shown are mean ± SE. Different letters indicate significant differences between depth or tree species at *p* < 0.05. E, *Eleiodoxa*; K, *Koompassia*; S, *Shorea**.

**Table 3:** The most abundant bacterial and archaeal phyla found in all samples from NSPSF with their respective relative abundance.

Phylum	Relative abundance of species (%)	Relative abundance of reads (%)
Proteobacteria	41.30	37.38
Acidobacteria	6.79	30.46
Verrucomicrobia	6.12	11.44
Planctomycetes	15.71	9.00
Bacteroidetes	7.60	2.35
Actinobacteria	3.62	1.51
Nitrospirae	0.51	1.33
Crenarchaeota	0.12	0.95
Euryarchaeota	0.79	3.53

On the other hand, the archaea in NSPSF showed relatively lower alpha diversity than Bacteria. Depth was found to significantly influence only the OTU richness (*F*_(2,60)_ = 6.758, *p* = 0.002) and Faith’s PD (*χ*^2^ (2) = 15.190, *p* < 0.001) – samples at 90 cm possessed significantly higher archaeal richness (*p* = 0.001) and Faith’s PD (*p* < 0.001) than surface samples (Table 2B). Tree species contributed significant impact only on the archaeal SDI (*χ*^2^ (2) = 8.8887, *p* = 0.0118) – *Eleiodoxa* demonstrated significantly lower archaeal diversity compared to *Shorea* (*p* = 0.003) (Table 2B).

### Microbial Community Composition

There were 20 bacterial phyla and 2 archaeal phyla found. Dominant phyla including Proteobacteria, Acidobacteria, Verrucomicrobia, and Planctomycetes, accounted for 88% of total sequences and 70% of total taxa in NSPSF (Table 3). Phyla Bacteroidetes, Actinobacteria, Nitrospirae, and Euryarchaeota were detected in lower abundance (>1% of total reads), whereas the other 14 phyla including Firmicutes and Crenarchaeota, were found in very low abundance (<1% of total reads).

**FIGURE 2:**
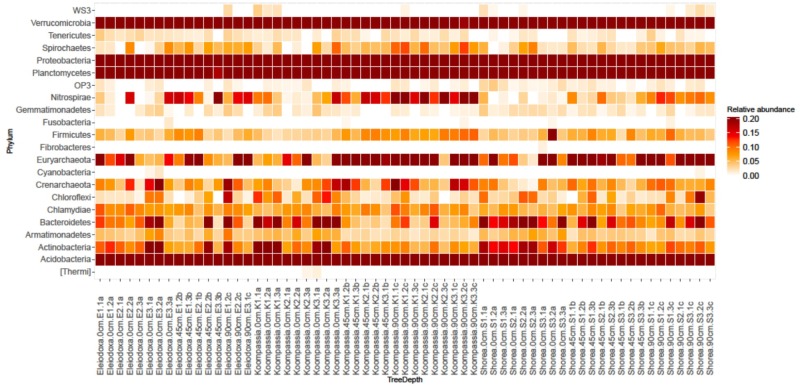
Heatmap showing relative abundance of phyla found in NSPSF according to tree species and depth (Dark red = high, white = low). Labels on the *x*-axis represent tree species.depth.replicate.

Among these phyla, Acidobacteria displayed significantly higher relative abundance in deeper layers, while both Planctomycetes and Actinobacteria showed the opposite trend (*p* < 0.05). At Class level, relative abundance of Betaproteobacteria was found to decrease significantly with depth, while Deltaproteobacteria showed significantly higher relative abundance in deeper layers (*p* < 0.05). The heatmap (Figure 2) displayed clear overview of the distribution of these phyla along the peat depth profile around each tree species. Phyla Proteobacteria, Acidobacteria, Verrucomicrobia and Planctomycetes were shown to be prevalent along the depth studied. Phyla Bacteroidetes and Actinobacteria were found more abundantly at the surface, whereas Nitrospirae and Euryarchaeota displayed higher relative abundance in deeper layers.

Microbial composition was studied up to the genus level and their relative abundance at different taxonomic ranks were reported (Supplementary Table S2). We found *Rhodoplanes* (Family Hyphomicrobiaceae, Order Rhodospirillales) as the most dominant genus in NSPSF. Regarding methane-related microbial taxa, the order E2 (Methanomassiliicoccales) affiliated to the class Thermoplasmata was the most prevalent methanogens found, and common methanogen groups, such as Methanomicrobiales and Methanosarcinales, were detected in negligible relative abundance. Methanotrophs were found in two groups: the family Methylocystaceae (type II methanotrophs) showed three orders of magnitude higher relative abundance than the family Methylococcaceae (type I methanotrophs). They displayed the highest relative abundance at the surface.

### Microbial Beta Diversity

**FIGURE 3:**
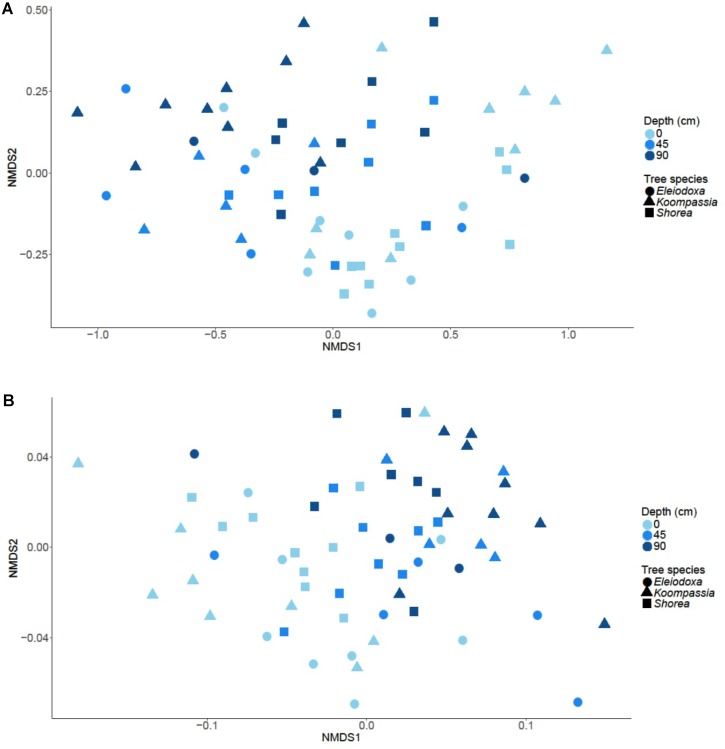
NMDS plot based on **(A)** Bray-Curtis dissimilarity and **(B)** weighted *UniFrac* distance reveal separation of microbial communities across depth in NSPSF.

Microbial composition was significantly distinct between depths (*p* = 0.001), but not tree species (*p* = 0.104). Depth explained about 27% of the compositional variation in the communities, whereas only 7% of the variation was due to the tree species. The NMDS plot of Bray-Curtis dissimilarity displayed separation of surface samples from samples at 90 cm, with samples at 45 cm overlapping mostly with the latter (Figure 3A). A similar pattern was observed in the NMDS plot using weighted *UniFrac* (Figure 3B), and microbial composition was again found to be significantly influenced by depths (*p* = 0.001) but not tree species (*p* = 0.517). The impact of depth on the microbial composition increased (31%) while the influence of tree species decreased (2%) in the latter analysis.

When the data were pooled according to depth, there was significant impact from tree species on the microbial composition at the surface (*p* = 0.013) and at 90 cm (*p* = 0.029) based on the Bray-Curtis dissimilarity. Tree species were responsible for 27% of microbial compositional variation at the surface and 34% of variation at 90 cm. In addition, according to the weighted *UniFrac*, microbial composition at 90 cm was significantly influenced by tree species (*p* = 0.015), which contributed 32% of microbial compositional variation. The same impact was, however, insignificant at the surface (*p* = 0.200) based on weighted *UniFrac*. The permutational ANOVA test showed significant dissimilarity on the microbial composition between depth (*p* < 0.001) and tree species (*p* < 0.001), as well as a significant interaction effect of tree species and depth (*p* = 0.015) on the microbial composition.

For the archaea, depth significantly impacted community composition based on Bray-Curtis dissimilarity (*p* < 0.001), accounting for 25% of the compositional variation found (Supplementary Figure S2A). The impact of depth decreased based on weighted *UniFrac* and became insignificant (*p* = 0.3300). Instead, tree species significantly affected the archaeal composition based on weighted *UniFrac* (*p* = 0.02), contributing 9% to the compositional variation found, although the plot (Supplementary Figure S2B) did not display apparent separation. Based on the permutational ANOVA test, the depth (*p* < 0.001) but not tree species (*p* = 0.051) significantly affected the observed variation among the communities, and there was no interaction effect of tree species and depth found (*p* = 0.139).

### Correlation Between Microbial Communities and Environmental Characteristics

Microbial community composition was found to be significantly correlated to six environmental variables studied (*p* < 0.001) – TN displayed the largest contribution (*r*^2^ = 0.3935), followed by DO (*r*^2^ = 0.3516), TPC (*r*^2^ = 0.3145), C/N ratio (*r*^2^ = 0.2987), and TP (*r*^2^ = 0.2829). Water pH (*r*^2^ = 0.1649, *p* = 0.006) was positively correlated with DO, TP and TN, and all of them correlated negatively with TPC and C/N ratio.

There were ten families under six phyla correlated significantly with TN (Supplementary Table S3). Relative abundance of almost all families correlated positively with TN (Acidobacteriaceae, Isosphaeraceae, Pirellulceae, Planctomycetaceae, Mycobacteriaceae, and Conexibacteraceae), except Syntrophobacteraceae, Alcaligenaceae, Thermodesulfovibrionaceae, and Nitrospiraceae. Consequently, relative abundance of five families from five phyla demonstrated significant correlation with C/N ratio – Alcaligenaceae and Solibacteraceae displayed relative abundance that formed positive correlation with C/N ratio, whereas relative abundance of families assigned to Planctomycetes and Actinobacteria showed a negative correlation to C/N ratio. Among these families, Acidobacteriaceae, Isosphaeraceae, Mycobacteriaceae and Conexibacteraceae were found to display significantly lower relative abundance in deeper layers, while both Syntrophobacteraceae and Alcaligenaceae demonstrated the opposite trend (*p* < 0.05).

A total of seven families under four phyla correlated significantly to depth and water pH, respectively. Relative abundance of families Isosphaeraceae, Planctomycetaceae, Mycobacteriaceae, Conexibacteraceae and Gaiellaceae displayed significant negative correlation to depth, whereas relative abundance of Syntrophobacteraceae and Methanomassiliicoccaceae were found to be significantly positively correlated to depth. In term of water pH, besides families Beijerinckiaceae and Syntrophobacteraceae, relative abundance of families belonged to phyla Planctomycetes and Actinobacteria formed positive correlation with water pH.

Only three families in three phyla established significant correlation to DO despite the anaerobiosis of the ecosystems (Syntrophobacteraceae: negative correlation; Mycobacteriaceae and Conexibacteraceae: positive correlation).

## Discussion

This study provides the first insight of microbial community structure along a depth profile around three abundant tree species in a tropical peat swamp forest located in Malaysia, using culture-independent next-generation sequencing. Similar studies are still rare apart from previous work conducted in the same North Selangor TPSF using enzymatic and DGGE approaches (Jackson et al., 2009), and studies conducted in TPSF in Thailand (Kanokratana et al., 2011) and Brunei (Tripathi et al., 2016) using NGS methods. Both vegetation (Thoms et al., 2010; Gömöryová et al., 2013; Ren et al., 2018) and depth (Eilers et al., 2012; Tsitko et al., 2014) have been reported to significantly impact on the belowground microbial community, and thus these hypotheses were tested in the extreme TPSF ecosystems.

### Depth, but Not Tree Species, Significantly Impacts Environmental Characteristics

The findings on the environmental characteristics in this study were typical of TPSF – extremely acidic, anoxic, nutrient-depleted but with high organic carbon and phenolic contents, and C/N ratio (Phillips, 1998; Page et al., 2006; Yule, 2010). TPSF in Southeast Asia are largely ombrotrophic, receiving water solely from the precipitation (no input from the river flow or underground water) (Yule, 2010). In addition, their constantly waterlogged conditions prevent the penetration of oxygen and lead to minimal organic carbon decomposition. Therefore, these ecosystems display extreme acidity, low oxygen and nutrients. Nutrient cycling occurs more rapidly at the surface where fresh leaf litter are available (Ong et al., 2015). Deeper layers are constituted of mostly the remnants of leaf litter decomposition, for example, the recalcitrant phenolic compounds such as lignin and tannin, and thus higher TPC was observed. Higher C/N ratio with increasing depth is attributed to nitrogen deficiency in deeper layers, as the organic carbon content was found to be homogenous along the depth profile. Reasons for homogenous organic carbon content could be (1) organic carbon is mostly in dissolved form (Gandois et al., 2014) and is redistributed under the waterlogged conditions, and (2) the dissolved organic compounds are actively consumed by microbial communities in deeper layers.

Contrary to our expectations, none of the environmental variables differed significantly between tree species. Studies have suggested that tree species is the main driver for distinct soil physicochemical properties such as pH, nutrient availability and C/N ratio (Binkley and Giardina, 1998; Thoms et al., 2010; Ushio et al., 2010). In addition, Ong et al. (2015) reported that *Koompassia* produces leaf litter of higher quality (higher nitrogen, phosphorus and lignin) than *Shorea* does. However, this study demonstrated that tree species does not leave a significant impact on the peat physicochemical properties in NSPSF. This could be due to several reasons such as (1) plants without nitrogen-fixers utilize the nitrogen exuded from the roots of legumes that has been fixed by nitrogen-fixing bacteria (Hoogmoed et al., 2014); (2) tree roots quickly penetrate leaf litter and recoup nutrients thus removing them from leaf litter (Ong et al., 2015); (3) the waterlogging and regular flooding of the forests redistribute nutrients.

### Depth, but Not Tree Species, Significantly Affects Microbial Diversity

NSPSF harbors high microbial diversity despite harsh environmental conditions. These microbial taxa are, however, dominated by only four major phyla – Proteobacteria, Acidobacteria, Verrucomicrobia and Planctomycetes, which are commonly found in peatland ecosystems (Kanokratana et al., 2011; Tsitko et al., 2014; Tripathi et al., 2016). The highest microbial diversity was detected at the surface, where substantial supply of newly senescent leaves sustain diverse microbial communities (Ong et al., 2015). Microbial diversity decreased following more extreme conditions in deeper layers. It is noteworthy that microbial diversity was found to be the lowest at 45 cm and increased at 90 cm. Similar results were reported in an acidic boreal peat bog in Finland (Tsitko et al., 2014) and in China (Zhou et al., 2017). It is still enigmatic as why exactly the microbial diversity decreased and increased again along the depth profile, but factors such as root networks and quality of organic matter should be considered.

Our results agreed with Hoogmoed et al. (2014) that tree species (whether associated with nitrogen-fixing bacteria or not) did not demonstrate significant impact on microbial diversity, probably because the environmental characteristics were similar across all tree species and thus providing similar niches for microbial communities. There were previous studies that suggested that the quality and composition of organic matter, rather than tree species, demonstrated direct impact on the bacterial diversity (Millard and Singh, 2010). The Bray-Curtis dissimilarity analysis showed minor influence of the tree species on microbial composition at the surface and at 90 cm. This could be due to distinct leaf litter quality and nutrient cycling mechanisms applied by different tree species at the surface (Ong et al., 2015); whereas at 90 cm, such observation may be due to diverse dissolved organic carbon and nitrogen in the root exudates (Smolander and Kitunen, 2002).

### Both Depth and Tree Species Significantly Affect the Archaeal Diversity

The archaeal OTU richness and Faith’s phylogenetic diversity (PD) increased significantly in deeper layers, largely because they thrive in anaerobiosis (Rampelotto, 2013). However, the archaeal Shannon diversity index (SDI) was found to decrease with depth albeit not significant. This result showed that while there were more archaeal taxa found in deeper layers, their abundance varied largely, indicating the presence of dominant taxa in deeper layers.

Regarding the impact of tree species on the archaeal diversity, the archaeal OTU richness and SDI increased in the order of *Eleiodoxa* < *Koompassia* < *Shorea*, possibly because huge buttress roots can produce root exudates that would sustain more diverse archaea. Their Faith’s PD were, however, similar across all tree species, indicating close phylogenetic relationship between the archaeal taxa. The result concurred with studies in northern peatlands (Rooney-Varga et al., 2007; Chanton et al., 2008), which showed that vegetation types influence archaeal activity and methanogenic pathways. However, the primer pairs used in this study were not specific for the archaea and thus might have caused the bias. Further investigation using archaea-specific primers would be essential for a better understanding on the archaeal communities in NSPSF.

### Interactive Effects From Environmental Variables Significantly Influence Microbial Community Structure

Dominance of Proteobacteria in the soil has been linked to rich carbon sources at the rhizosphere (Fierer et al., 2007). The family Rhodospirillaceae was noted for performing fermentation utilizing formate, butyrate, lactate, and propionate (Hunger et al., 2011; Hausmann et al., 2016). The most abundant genus, *Rhodoplanes*, are likely involved in nitrogen fixation (Pershina et al., 2015), which corresponded with the finding that total nitrogen was the dominant abiotic factor influencing microbial communities in NSPSF. Consequently, it is believed that nitrogen fixation could be one of the most prevalent biogeochemical processes occur in TPSF. This is reinforced by the fact that TPSF support enormous plant diversity and diverse microbial communities (Yule, 2010). In such a nutrient-depleted environment, nitrogen-fixing bacteria are essential to ensure sufficient nutrient supply and maintain the functions of these thickly forested ecosystems.

Generally, pH has been reported as the main driver of microbial diversity (Fierer et al., 2012; Elliott et al., 2015; Zhang et al., 2015; Tripathi et al., 2016; Ren et al., 2018). Our results denoted its significant contribution in microbial composition in NSPSF, albeit not the most prominent factor. It is likely that abiotic factors other than pH also contribute equally, if not stronger to shape microbial communities in TPSF, for instance, oxygen availability, soil moisture and nitrogen content (Ren et al., 2018). It is noteworthy that the interactive effects from various environmental variables contribute concurrently and leave significant impact on microbial diversity and composition in TPSF. As shown in correlation tests in this study, relative abundance of microbial groups responded significantly to >1 environmental variable, mainly because relationships between biotic and abiotic factors in natural ecosystems are generally complex to guarantee the ecosystem functioning and sustainability. There are commonly strong correlations between microbial communities and physicochemical properties of peat, but further investigation is required to elucidate the complete framework.

The results are useful indicators for the favored environmental characteristics of various microbial groups, for instance, Syntrophobacteraceae showed significantly higher relative abundance with depth, and displayed significantly negative correlation to water pH, DO, and TN, indicating that this family favored acidic, anaerobic and nutrient depleted habitats (Ward et al., 2009; Pankratov et al., 2011; Tsitko et al., 2014). Members of Planctomycetes have mostly been known as aerobic chemoorganotrophic (Ivanova and Dedysh, 2006), but this study showed that DO was not the main factor for their growth, and they might not be solely obligate aerobes. They favored upper peat layers, where more labile substrates were available and higher nitrogen supply was guaranteed.

Euryarchaeota showed significantly positive correlation to TPC, which might indicate the possibility of this community in utilizing phenolic compounds. The relative abundance of its largest family, Methanomassiliicoccaceae, did not show significant correlation to DO, although they are known as strictly anaerobes. It is concurred with previous studies that they were detected also at the surface peat (Isanapong et al., 2012; Kotak et al., 2015), which is not completely anoxic. This could be due to the reasons such as (i) some methanogens are only inhibited, but not killed by oxygen availability (Yavitt et al., 2006), (ii) active consumption of oxygen by aerobic bacteria at the surface helps to prevent complete inhibition on methanogenesis and enables both aerobic and anaerobic microbes grow in parallel (Tholen et al., 2007).

### Comparison in Microbial Community Structure Between NSPSF With Other Peatlands

In comparison with a similar study conducted by Jackson et al. (2009) in NSPSF, our results agreed that microbial composition differed significantly with depths. However, findings that disagreed with the current results included (i) Acidobacteria was most abundant (27–54%), (ii) the archaea were detected only in deeper layers (20–50 cm) and all belonged to Crenarchaeota, (iii) No methanogens were found (Jackson et al., 2009). These differences are likely due to different primers and methodologies used, as they applied DGGE and enzymatic techniques. NSPSF shared similar microbial diversity with a Thai TPSF (Kanokratana et al., 2011) despite distinct pH (Thai TPSF = pH 5), but was different to a TPSF in Brunei (pH 3.4). This again proved that pH is not necessarily the main driver in microbial diversity in TPSF. In addition, biogeography could also be an underlying explanation.

In comparison with northern peatlands, Acidobacteria rather than Proteobacteria was the dominant phylum in boreal peat bogs in Finland (Tsitko et al., 2014) and Russia (Serkebaeva et al., 2013), and in temperate peat bogs in Minnesota (Lin et al., 2012) and Czech Republic (Urbanová and Bárta, 2014). Proteobacteria and Verrucomicrobia were also found abundantly in these peatlands. In addition, these peatlands, harbored significantly abundant Firmicutes, the key players in cellulose degradation, which favor neutral habitats (Pankratov et al., 2006). Firmicutes were found in very low abundance in NSPSF, possibly due to lower pH in NSPSF compared to a pH range of 4–6 for northern peatlands (Lin et al., 2012). The fact that Thai peat swamp forest with pH 5 also detected abundant Firmicutes (Kanokratana et al., 2011) reinforced the significant effect of pH instead of climate on Firmicutes.

With regards to methane-related microbes, a majority of the methanogens from NSPSF belonged to the order E2, which is currently recognized as Methanomassiliicoccales under the class Thermoplasmata (Iino et al., 2013). Methanogens were generally documented to be most abundant at the oxic-anoxic interface (Cadillo-Quiroz et al., 2006; Lipson et al., 2013). However, their relative abundance was found to increase with depth in NSPSF albeit insignificant statistically. A similar result was also reported in a Finnish oligotrophic fen (Galand et al., 2002). They likely consume dissolved organic materials from young upper peat layers (Gandois et al., 2014). Kanokratana et al. (2011) detected methanogens from the order Methanomicrobiales in a Thai TPSF, which were found in extreme low abundance in NSPSF. Methanomicrobiales are hydrogenotrophic methanogens reducing CO_2_ using H_2_ as electron acceptor to produce CH_4_, whereas Methanomassiliicoccales are obligately methylotrophic methanogens, which produce CH_4_ through one-carbon compounds, for example, methanol reduction using H_2_ as an electron acceptor (Iino et al., 2013). Methanol is commonly produced during anaerobic decomposition of plant tissues (Schink and Zeikus, 1980) and thus TPSF become methane sources. It is noteworthy that there is taxonomic confusion regarding these two methanogenic orders, as E2 has been reported as a component of Methanomicrobiales and sequences from peatlands in Finland, Germany, Canada, and United Kingdom were found to cluster within group E2 under Methanomicrobiales (Cadillo-Quiroz et al., 2006). Possible explanation could be that the class Thermoplasmata was not included and thus the resolution of the phylogenetic tree was hampered. In term of methanotrophs, higher relative abundance of Methylocystaceae was detected in NSPSF compared to Methylococcaceae concurred with findings in northern peatlands (Dedysh, 2002; Lin et al., 2012, 2014), which could be attributed to the following reasons: (i) Methylocystaceae are favored in acidic environments (Lin et al., 2014) and (ii) Methylocystaceae are capable of nitrogen fixation (Bao et al., 2014), which allows them to thrive in nutrient-depleted environments.

## Conclusion

An increasing interest has emerged regarding microbial ecology in tropical peatlands following the recognition of their prominent role in global carbon cycle and climate change. To the best of our knowledge, this is the first culture-independent metabarcoding study investigating the impact of tree species and depth on the microbial diversity and composition in a Southeast Asian TPSF. This study illustrated significant role depth plays in microbial community structure, and thus we recommend the inclusion of this factor in future microbial ecological research in peatlands ecosystems. Tree species might play an important role be influencing microbial diversity and composition, but to a less extent than expected in tropical peatlands. More environmental characteristics, such as dissolved organic matter and ammonium content, should also be included, as the interactive effects from environmental variables are responsible for microbial community structure along the depth profile. In addition, microbial species databases need to be improved to give a better resolution. Bacterial cultivation techniques should also be developed in parallel with biochemical assays for characterization of potentially novel species. With known microbial diversity in TPSF, studies focusing on specific microbial groups (e.g., nitrogen-fixing bacteria, methanogens and methanotrophs) or functional traits/genes (*nif, mmo*) are highly recommended to understand their involvement in peat formation, nutrient cycling, biogeochemical processes and climate change. TPSF are absolutely under-explored microbial pools, and similar research should be conducted in larger scale in order to generate better insights on the current rudimentary knowledge of microbial ecology in these endangered ecosystems.

## Author Contributions

CY, SL, and AK designed the study. CT and CY conducted sample collection. CT performed the experiments for environmental parameters, data analyses, and drafted the manuscript. CT and WS performed molecular work. AK performed bioinformatics. All authors contributed to the final version of the manuscript.

## Conflict of Interest Statement

The authors declare that the research was conducted in the absence of any commercial or financial relationships that could be construed as a potential conflict of interest.

## Footnotes

1 https://www.ebi.ac.uk/ena/
