# Supplementary material for: Microbial Community Structure in a Malaysian Tropical Peat Swamp Forest: The Influence of Tree Species and Depth
Source: Front Microbiol. 2018 Dec 4;9:2859. doi: 10.3389/fmicb.2018.02859 (PMC6288306; doi:10.3389/fmicb.2018.02859)
Supplement: Supplementary file 1 [file Table_1.docx]

Table S1

The environmental characteristics according to tree species and depths in NSPSF. All values shown are mean ± SE. DO: dissolved oxygen; TN: total nitrogen; TP: total phosphorus; TPC: total phenolic content.

| **Tree species and depth** | **Water pH** | **Peat pH** | | **Organic carbon content**  **(%)** | **DO (mg/ℓ)** | **TN (%)** | **TP (µg/g)** | **TPC**  **(mg TAE/ g)** | **C/ N ratio** |
| --- | --- | --- | --- | --- | --- | --- | --- | --- | --- |
|  |  | **H_2_O** | **CaCl_2_** |  |  |  |  |  |  |
| *Eleiodoxa*  0 cm  45 cm  90 cm | 3.45 ± 0.05  3.40 ± 0.08  3.09 ± 0.02 | 3.41 ± 0.11  3.29 ± 0.08  3.35 ± 0.01 | 2.72 ± 0.17  2.52 ± 0.06  2.50 ± 0.04 | 55.23 ± 0.37  56.25 ± 0.34  56.51 ± 0.30 | 1.63 ± 0.03  0.80 ± 0.09  0.56 ± 0.03 | 1.93 ± 0.20  1.60 ± 0.09  1.17 ± 0.14 | 214.27 ± 9.03  128.57 ± 8.29  53.78 ± 9.67 | 78.99 ± 26.89  213.00 ± 43.54  276.29 ± 46.59 | 29.28 ± 3.03  35.41 ± 2.22  49.71 ± 5.79 |
| *Koompassia*  0 cm  45 cm  90 cm | 3.53 ± 0.09  3.35 ± 0.20  3.14 ± 0.20 | 3.44 ± 0.03  3.62 ± 0.16  3.43 ± 0.09 | 2.71 ± 0.03  2.75 ± 0.16  2.65 ± 0.11 | 55.67 ± 0.58  54.10 ± 0.74  55.71 ± 0.87 | 1.63 ± 0.03  1.05 ± 0.11  0.77 ± 0.15 | 1.85 ± 0.07  1.56 ± 0.09  1.12 ± 0.23 | 230.77 ± 19.96  151.90 ± 19.98  103.13 ± 16.76 | 117.30 ± 2.94  213.82 ± 5.61  226.43 ± 39.49 | 30.12 ± 1.09  34.92 ± 2.64  55.17 ± 13.05 |
| *Shorea*  0 cm  45 cm  90 cm | 3.49 ± 0.05  3.29 ± 0.09  3.28 ± 0.11 | 3.41 ± 0.11  3.51 ± 0.06  3.36 ± 0.17 | 2.92 ± 0.21  2.70 ± 0.06  2.55 ± 0.18 | 55.61 ± 0.49  55.07 ± 0.53  55.95 ± 0.86 | 1.67 ± 0.07  1.01 ± 0.17  0.59 ± 0.02 | 2.12 ± 0.15  1.73 ± 0.09  1.32 ± 0.24 | 224.23 ± 4.60  146.07 ± 10.40  65.54 ± 40.73 | 119.83 ± 35.58  198.12 ±14.78  243.97 ± 52.38 | 26.55 ± 1.22  32.07 ± 1.21  45.10 ± 4.84 |
