# Supplementary material for: Microbial Community Structure in a Malaysian Tropical Peat Swamp Forest: The Influence of Tree Species and Depth
Source: Front Microbiol. 2018 Dec 4;9:2859. doi: 10.3389/fmicb.2018.02859 (PMC6288306; doi:10.3389/fmicb.2018.02859)
Supplement: Supplementary file 2 [file Table_2.docx]

Table S2. Microbial community composition in NSPSF showing major taxa in each taxonomic rank. Percentages in brackets dedicated their relative abundance in previous taxonomic rank (e.g. Alphaproteobacteria comprised 54.08% of total reads affiliated to Proteobacteria).

| **Phylum** | **Class** | **Order** | **Family** | **Genus** |
| --- | --- | --- | --- | --- |
| Proteobacteria (37.38%) | Alphaproteobacteria (54.08%) | Rhodospirillales (48.32%) | Rhodospirillaceae (81.64%) | Unknown (98.32%) |
|  |  |  | Acetobacteraceae (18.36%) | *Acidocella* (4.79%) |
|  |  |  |  | Unknown (94.55%) |
|  |  | Rhizobiales (46.29%) | Hyphomicrobiaceae (78.80%) | *Rhodoplanes* (96%) |
|  |  |  |  | *Devosia* (1.12%) |
|  |  |  | Methylocystaceae (15.43%) | *Methylosinus* (1.19%) |
|  |  |  |  | *Pleomorphomonas*(0.89%) |
|  |  |  |  | Unknown (97.92%) |
|  |  |  | Beijerinckiaceae (4.06%) | Unknown (100%) |
|  |  | Caulobacterales (3.36%) | Caulobacteraceae (100%) | *Phenylobacterium* (22.10%) |
|  |  |  |  | Unknown (75.37%) |
|  |  |  |  |  |
|  | Deltaproteobacteria (20.92%) | Syntrophobacterales (76.18%) | Syntrophobacteraceae (90.36%) | *Syntrophobacter* (16.77%) |
|  |  |  |  | Unknown (83.23%) |
|  |  |  | Syntrophorhabdaceae (6.38%) | Unknown (100%) |
|  |  |  | Syntrophaceae (3.26%) | *Desulfobacca* (98.28%) |
|  |  |  |  | *Desulfomonile* (1.72%) |
|  |  | Myxococcales (14.74%) | Haliangiaceae (28.63%) | Unknown (100%) |
|  |  |  | Polyangiaceae (24.59%) | Unknown (99.98%) |
|  |  |  | Myxococcaceae (40.25%) | *Anaeromyxobacter* (22.82%) |
|  |  |  |  | Unknown (77.18%) |
|  |  | Bdellovibrionales (6.12%) | Bdellovibrionaceae (96.36%) | *Bdellovibrio* (95.78%) |
|  |  |  | Bacteriovoracaceae (3.64%) | Unknown (98.96%) |
|  |  |  |  |  |
|  | Gammaproteobacteria (17.08%) | Xanthomonadales (86.34%) | Sinobacteraceae (91.29%) | Unknown (98.06%) |
|  |  |  | Xanthomonadaceae (8.71%) | Unknown (87.64%) |
|  |  | Legionellales (9.17%) | Coxiellaceae (88.95%) | *Aquicella* (37.33%) |
|  |  |  |  | Unknown (61.58%) |
|  |  |  | Legionellaceae (11.05%) | *Legionella* (30.59%) |
|  |  |  |  | Unknown (63.46%) |
|  |  | Methylococcales (0.17%) | Methylococcaceae (100%) | *Methylomonas* (88.24%) |
|  |  |  |  | Unknown (23.16%) |
|  |  |  |  |  |
|  | Betaproteobacteria (7.92%) | Burkholderiales (87.47%) | Burkholderiaceae (55.67%) | *Burkholderia* (46.77%) |
|  |  |  |  | *Salinispora* (44.25%) |
|  |  |  |  | Unknown (9%) |
|  |  |  | Alcaligenaceae (23.23%) | *Achromobacter* (1.36%) |
|  |  |  |  | Unknown (98.64%) |
|  |  |  | Comamonadaceae (8.89%) | Unknown (92.14%) |
|  |  |  | Oxalobacteraceae (12.21%) | *Janthinobacterium* (14.29%) |
|  |  |  |  | Unknown (81.67%) |
|  |  | Neisseriales (7.8%) | Neisseriaceae (100%) | Unknown (99.64%) |
|  |  |  |  |  |
| Acidobacteria (30.46%) | Acidobacteriia (77.10%) | Acidobacteriales (100%) | Acidobacteriaceae (26.54%) | Unknown (99.01%) |
|  |  |  | Koribacteraceae (73.46%) | *Candidatus Koribacter* (7.85%) |
|  |  |  |  | Unknown (92.15%) |
|  | Solibacteres (19.15%) | Solibacterales (100%) | Solibacteraceae (100%) | *Candidatus Solibacter* (87.75%) |
|  |  |  |  |  |
| Verrucomicrobia (11.44%) | Pedosphaerae (76.71%) | Pedosphaerales (100%) | Pedosphaeraceae (5.52%) | *Pedosphaera* (5.35%) |
|  |  |  |  | Unknown (94.65%) |
|  |  |  | auto67_4W (84.38%) | Unknown (100%) |
|  |  |  | Ellin515 (8.92%) | Unknown (100%) |
|  | Opitutae (12.41%) | Opitutales (100%) | Opitutaceae (100%) | *Opitutus* (90.46%) |
|  |  |  |  | Unknown (9.54%) |
|  | Spartobacteria (10.38%) | Chthoniobacterales (100%) | Chthoniobacteraceae (100%) | *Candidatus Xiphinematobacter* (38.88%) |
|  |  |  |  | *Chthoniobacter* (22.8%) |
|  |  |  |  | Unknown (27.97%) |
|  |  |  |  |  |
| Planctomycetes (9%) | Plantomycetia (99.95%) | Gemmatales (71.3%) | Gemmataceae (58.44%) | *Gemmata* (15.47%) |
|  |  |  |  | Unknown (84.53%) |
|  |  |  | Isosphaeraceae (41.56%) | Unknown (99.98%) |
|  |  | Pirellulales (14.39%) | Pirellulaceae (100%) | A17 (17.11%) |
|  |  |  |  | Unknown (82.20%) |
|  |  | Planctomycetales (14.26%) | Planctomycetaceae (100%) | Planctomyces (100%) |
|  |  |  |  |  |
| Bacteroidetes (2.35%) | Saprospirae (72.86%) | Saprospirales (100%) | Chitinophagaceae (99.51%) | *Chitinophaga* (1.02%) |
|  |  |  |  | Unknown (98.16%) |
|  | Cytophagia (16.94%) | Cytophagales (100%) | Cytophagaceae (96.70%) | *Cytophaga* (8.36%) |
|  |  |  |  | Unknown (88.55%) |
|  | Sphingobacteriia (5.81%) | Sphingobacteriales (100%) | Sphingobacteriaceae (100%) | *Pedobacter* (15.53%) |
|  |  |  |  | *Sphingobacterium* (3.99%) |
|  |  |  |  | Unknown (80.48%) |
|  |  |  |  |  |
| Actinobacteria (1.51%) | Actinobacteria (62.36%) | Actinomycetales (97.63%) | Mycobacteriaceae (49.70%) | *Mycobacterium* (100%) |
|  |  |  | Thermomonosporaceae (8.05%) | *Actinoallomurus* (88.20%) |
|  |  |  |  | Unknown (9.09%) |
|  |  |  | Streptomycetaceae (7.88%) | *Streptomyces* (16.76%) |
|  |  |  |  | Unknown (83.24%) |
|  |  |  | Micromonosporaceae (7.68%) | *Pilimelia* (13.73%) |
|  |  |  |  | *Dactylosporagium* (9.29%) |
|  |  |  |  | Unknown (70.82%) |
|  | Thermoleophilia (35.52%) | Solirubrobacteriales (53.94%) | Conexibacteraceae (94.83%) | *Conexibacter* (60.04%) |
|  |  |  |  | Unknown (39.96%) |
|  |  |  | Solirubrobacteraceae (4.81%) | Unknown (100%) |
|  |  | Gaiellales (46.06%) | Gaiellaceae (99.33%) | Unknown 993.33%) |
|  |  |  |  |  |
| Nitrospirae (1.33%) | Nitrospira (100%) | Nitrospirales (100%) | Thermodesulfovibrionaceae (78.08%) | Unknown (72.86%) |
|  |  |  | Nitrospiraceae (4.95%) | *Nitrospira* (78.6%) |
|  |  |  |  | Unknown (21.40%) |
|  |  |  |  |  |
| Crenarchaeota (0.95%) | Thaumarchaeota (100%) | Cenarchaeales (54.89%) | Cenarchaeaceae (0.02%) |  |
|  |  |  | SAGMA-X (99.98%) |  |
|  |  | Nitrososphaerales (45.11%) | Nitrososphaeraceae (100%) | *Candidatus Nitrososphaera* (100%) |
|  |  |  |  |  |
| Euryarchaeota (3.53%) | Thermoplasmata (99.12%) | E2 (99.58%) | Methanomassiliicoccaceae (98.70%) | Unknown (98.80%) |
|  | Methanomicrobia (0.48%) | Methanocellales (79.64%) | Methanocellaceae (100%) | *Methanocella*(100%) |
|  |  | Methanomicrobiales (10.55%) | Methanoregulaceae (65.525) | *Candidatus Methanoregula* (100%) |
|  |  |  | Methanospirillaceae (34.48%) | *Methanospirillum* (100%) |
|  |  | Methanosarcinales (9.81%) | Methanosarcinaceae (100%) | *Methanosarcina* (100%) |
|  | Methanobacteria (0.4%) | Methanobacteriales (100%) | Methanobacteriaceae (11.27%) | *Methanobacterium* (16.20%) |
|  |  |  | MSBL1 (88.73%) | SAGMEG-1(100%) |
