# Supplementary material for: Microbial Community Structure in a Malaysian Tropical Peat Swamp Forest: The Influence of Tree Species and Depth
Source: Front Microbiol. 2018 Dec 4;9:2859. doi: 10.3389/fmicb.2018.02859 (PMC6288306; doi:10.3389/fmicb.2018.02859)
Supplement: Supplementary file 3 [file Table_3.docx]

Table S3

Correlation between relative abundance of major phyla/ classes/ orders/ families and environmental characteristics based on Pearson’s or Spearman’s rank correlation coefficients. Values in bold are significant at *p* < 0.05, values in bold * are significant at *p* < 0.01. DO: dissolved oxygen; TN: total nitrogen; TP: total phosphorus; TPC: total phenolic content.

| **Phyla/ Classes/ Orders/ Families** | **Depth** | **Water pH** | **DO** | **TN** | **TP** | **TPC** | **C/N ratio** |
| --- | --- | --- | --- | --- | --- | --- | --- |
| Proteobacteria | - 0.251 | 0.317 | 0.134 | 0.108 | 0.200 | - 0.333 | - 0.048 |
| Alphaproteobacteria | - 0.335 | 0.333 | 0.159 | 0.331 | 0.200 | - 0.391 | - 0.302 |
| Rhodospirillales | 0.003 | - 0.100 | - 0.167 | 0.182 | - 0.283 | - 0.001 | - 0.192 |
| Rhodospirillaceae | 0.483 | - 0.381 | - 0.604 | - 0.276 | - 0.651 | 0.547 | 0.211 |
| Acetobacteraceae | - 0.556 | 0.512 | 0.518 | 0.621 | 0.455 | - 0.640 | - 0.558 |
| Rhizobiales | - 0.273 | 0.167 | 0.067 | 0.219 | 0.083 | - 0.392 | - 0.181 |
| Hyphomicrobiaceae | - 0.221 | 0.196 | 0.248 | 0.186 | 0.127 | - 0.341 | - 0.150 |
| Methylocystaceae | - 0.384 | 0.170 | 0.294 | 0.338 | 0.222 | - 0.418 | - 0.303 |
| Beijerinckiaceae | 0.619 | **- 0.669** | - 0.632 | - 0.622 | - 0.659 | 0.544 | 0.605 |
| Deltaproteobacteria | **0.755** | **- 0.767** | **- 0.711** | **- 0.900**^*^ | **- 0.700** | **0.713** | **0.867**^*^ |
| Syntrophobacterales | 0.553 | - 0.617 | **- 0.711** | - 0.579 | **- 0.700** | 0.359 | **0.717** |
| Syntrophobacteraceae | **0.709** | **-0.705** | **- 0.699** | **- 0.706** | - 0.641 | **0.677** | 0.649 |
| Myxococcales | - 0.230 | 0.178 | 0.183 | 0.164 | 0.120 | - 0.172 | - 0.113 |
| Myxococcaceae | - 0.087 | - 0.026 | 0.022 | - 0.035 | 0.001 | - 0.072 | 0.088 |
| Haliangiaceae | - 0.007 | - 0.011 | 0.007 | - 0.015 | - 0.073 | 0.038 | 0.058 |
| Polyangiaceae | - 0.516 | 0.464 | 0.416 | 0.465 | 0.385 | - 0.452 | - 0.416 |
| Bdellovibrioceae | - 0.488 | 0.465 | 0.572 | 0.518 | 0.468 | - 0.530 | - 0.438 |
| Gammaproteobacteria | - 0.561 | 0.517 | 0.427 | 0.549 | 0.467 | - 0.491 | - 0.504 |
| Xanthomonadales | - 0.587 | 0.567 | 0.477 | 0.585 | 0.517 | - 0.506 | - 0.544 |
| Sinobacteraceae | - 0.571 | 0.466 | 0.513 | 0.591 | 0.443 | - 0.480 | - 0.558 |
| Xanthomonadaceae | - 0.512 | 0.399 | 0.546 | 0.421 | 0.493 | - 0.518 | - 0.338 |
| Coxiellaceae | - 0.226 | 0.177 | 0.299 | 0.133 | 0.248 | - 0.231 | - 0.077 |
| Legionellaceae | 0.195 | - 0.376 | - 0.280 | - 0.108 | - 0.297 | 0.222 | 0.109 |
| Betaproteobacteria | - 0.541 | 0.583 | 0.410 | 0.420 | 0.450 | - 0.623 | - 0.335 |
| Burkholderiales | - 0.362 | - 0.450 | 0.427 | 0.466 | 0.467 | - 0.333 | - 0.452 |
| Burkholderiaceae | - 0.623 | 0.573 | 0.574 | 0.595 | 0.510 | - 0.634 | - 0.535 |
| Alcaligenaceae | 0.653 | - 0.557 | - 0.596 | **- 0.800*** | - 0.576 | 0.631 | **0.772** |
| Oxalobacteraceae | - 0.377 | 0.284 | 0.465 | 0.353 | 0.385 | - 0.547 | - 0.239 |
| Comamonadaceae | - 0.243 | 0.288 | 0.228 | 0.160 | 0.186 | - 0.250 | - 0.100 |
| Neisseriaceae | 0.577 | - 0.496 | - 0.453 | - 0.528 | - 0.492 | 0.321 | 0.532 |
| Acidobacteria | 0.445 | - 0.383 | - 0.326 | - 0.378 | - 0.367 | 0.350 | 0.316 |
| Acidobacteriales | 0.425 | - 0.450 | - 0.343 | - 0.357 | - 0.383 | 0.448 | 0.299 |
| Koribacteraceae | 0.573 | - 0.519 | - 0.625 | - 0.546 | - 0.519 | 0.600 | 0.477 |
| Acidobacteriaceae | - 0.541 | 0.548 | 0.514 | **0.710** | 0.491 | - 0.574 | **- 0.679** |
| Solibacterales | 0.489 | - 0.650 | **- 0.678** | - 0.638 | **- 0.733** | 0.289 | **0.717** |
| Solibacteraceae | 0.274 | -0.224 | - 0.460 | - 0.257 | - 0.406 | 0.319 | **0.231** |
| Verrucomicrobia | - 0.506 | 0.417 | 0.460 | **0.676** | 0.439 | - 0.431 | - 0.685 |
| Pedosphaeraceae | - 0.531 | 0.395 | 0.458 | 0.565 | 0.385 | - 0.416 | - 0.539 |
| Opitutales | - 0.485 | 0.367 | 0.393 | 0.496 | 0.333 | - 0.488 | - 0.446 |
| Opitutaceae | - 0.485 | 0.299 | 0.472 | 0.496 | 0.367 | - 0.488 | - 0.446 |
| Chthoniobacteraceae | - 0.583 | 0.579 | 0.517 | 0.603 | 0.476 | - 0.463 | - 0.565 |
| Planctomycetes | **- 0.785** | **0.700** | 0.628 | **0.780**^*^ | **0.683** | - 0.715 | **- 0.783** |
| Gemmatales | **- 0.781** | **0.683** | 0.594 | **0.783** | 0.633 | **-0.718**^*^ | **- 0.733** |
| Gemmataceae | - 0.656 | 0.467 | 0.565 | 0.567 | 0.531 | - 0.564 | - 0.558 |
| Isosphaeraceae | **- 0.721** | **0.689** | 0.624 | **0.711** | 0.651 | **- 0.715** | **- 0.803*** |
| Pirellulaceae | - 0.478 | 0.355 | 0.472 | **0.473** | 0.399 | - 0.451 | - 0.426 |
| Planctomycetaceae | **- 0.689** | **0.693** | 0.659 | **0.771** | 0.626 | - 0.623 | **- 0.742** |
| Bacteroidetes | - 0.305 | 0.383 | 0.251 | 0.296 | 0.300 | - 0.338 | - 0.228 |
| Chitinophagaceae | - 0.393 | 0.296 | 0.420 | 0.384 | 0.327 | - 0.416 | - 0.314 |
| Cytophagales | - 0.061 | 0.050 | - 0.109 | 0.076 | - 0.050 | - 0.106 | - 0.015 |
| Cytophagaceae | - 0.058 | 0.067 | 0.083 | 0.069 | - 0.021 | - 0.104 | - 0.007 |
| Sphingobacteriaceae | - 0.518 | 0.426 | 0.543 | 0.448 | 0.483 | - 0.589 | - 0.352 |
| Actinobacteria | **- 0.709** | **0.700** | 0.569 | **0.75** | 0.600 | - 0.651 | **- 0.700** |
| Actinomycetales | **- 0.797** | **0.683** | 0.594 | **0.761** | 0.583 | **- 0.730** | **- 0.733** |
| Mycobacteriaceae | **- 0.894*** | **0.851*** | **0.834*** | **0.920*** | **0.831*** | **- 0.846*** | **- 0.882*** |
| Conexibacteraceae | **- 0.779** | **0.714** | **0.734** | **0.692** | **0.690** | **- 0.768** | - 0.636 |
| Solirubrobacteraceae | - 0.019 | 0.040 | 0.069 | 0.011 | 0.012 | - 0.121 | 0.068 |
| Gaiellaceae | **- 0.474** | **0.368** | 0.440 | 0.384 | 0.360 | - 0.419 | - 0.358 |
| Nitrospirae | 0.637 | - 0.467 | - 0.544 | **- 0.771** | - 0.533 | 0.570 | **0.750** |
| Thermodesulfovibrionaceae | 0.598 | - 0.538 | - 0.629 | **- 0.686** | - 0.583 | 0.529 | 0.618 |
| Nitrospiraceae | 0.394 | - 0.343 | - 0.181 | **- 0.510** | - 0.300 | 0.247 | 0.551 |
| Euryarchaeota | **0.697** | **- 0.700** | - 0.544 | - 0.577 | - 0.600 | **0.733** | 0.526 |
| Methanomassiliicoccaceae | **0.689** | - 0.556 | -0.612 | - 0.566 | - 0.606 | 0.648 | 0.514 |
| Crenarchaeota | 0.411 | - 0.517 | - 0.402 | - 0.608 | - 0.350 | 0.253 | 0.603 |
| SAGMA-X | 0.447 | - 0.559 | - 0.333 | - 0.657 | - 0.397 | 0.297 | 0.648 |
| Nitrososphaeraceae | - 0.273 | 0.285 | 0.377 | 0.284 | 0.389 | - 0.338 | - 0.310 |
