# Supplementary figures and images for: Microbial Community Structure in a Malaysian Tropical Peat Swamp Forest: The Influence of Tree Species and Depth
Source: Front Microbiol. 2018 Dec 4;9:2859. doi: 10.3389/fmicb.2018.02859 (PMC6288306; doi:10.3389/fmicb.2018.02859)

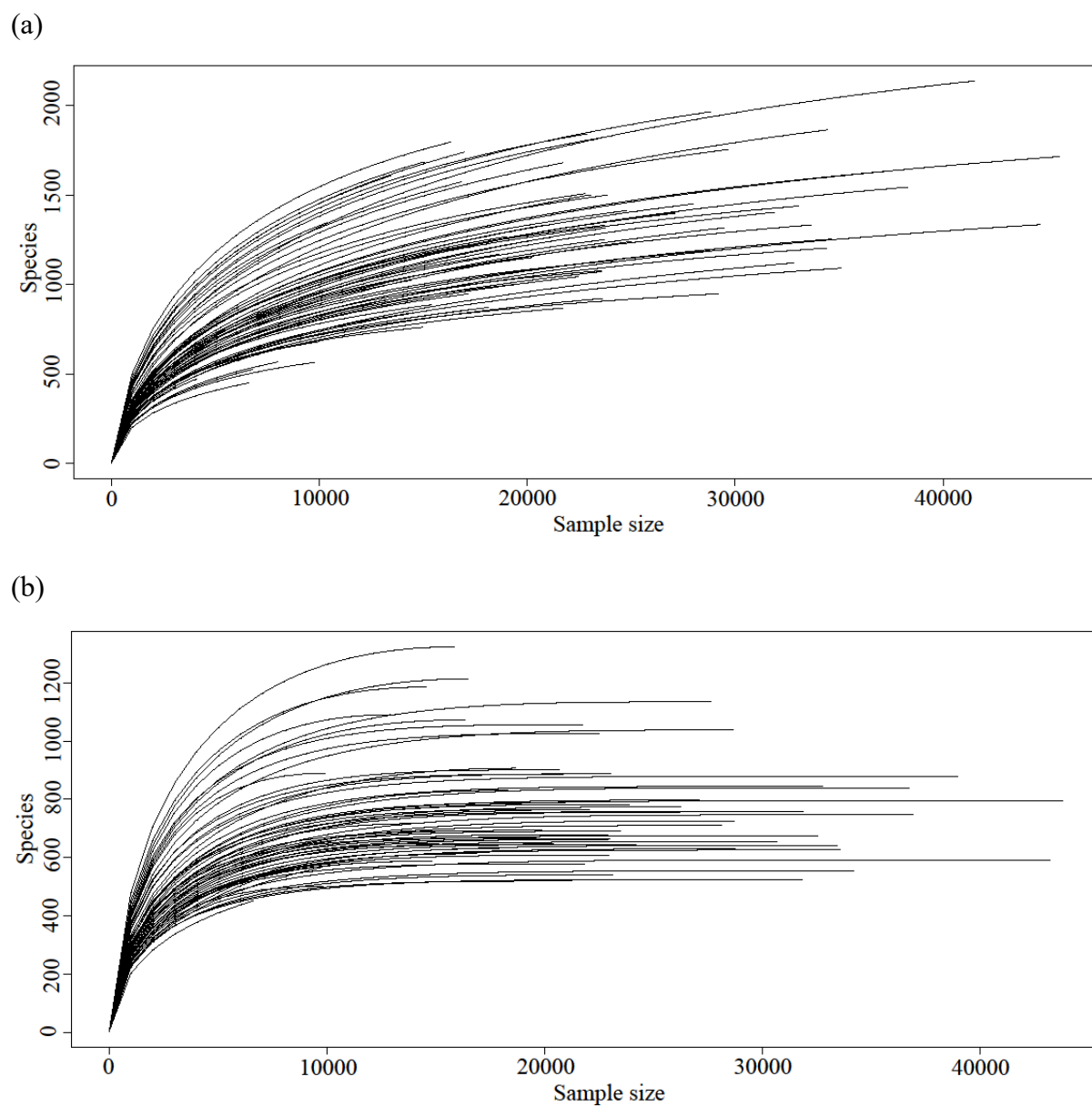

Figure S1. Rarefaction curves for (a) unfiltered taxa and (b) 0.01% filtered taxa.

Supplement: Supplementary file 4 [file Image_1.pdf]
