# Supplementary material for: Microbial Community Structure in a Malaysian Tropical Peat Swamp Forest: The Influence of Tree Species and Depth
Source: Front Microbiol. 2018 Dec 4;9:2859. doi: 10.3389/fmicb.2018.02859 (PMC6288306; doi:10.3389/fmicb.2018.02859)
Supplement: Supplementary file 5 [file Image_2.pdf]

(a)

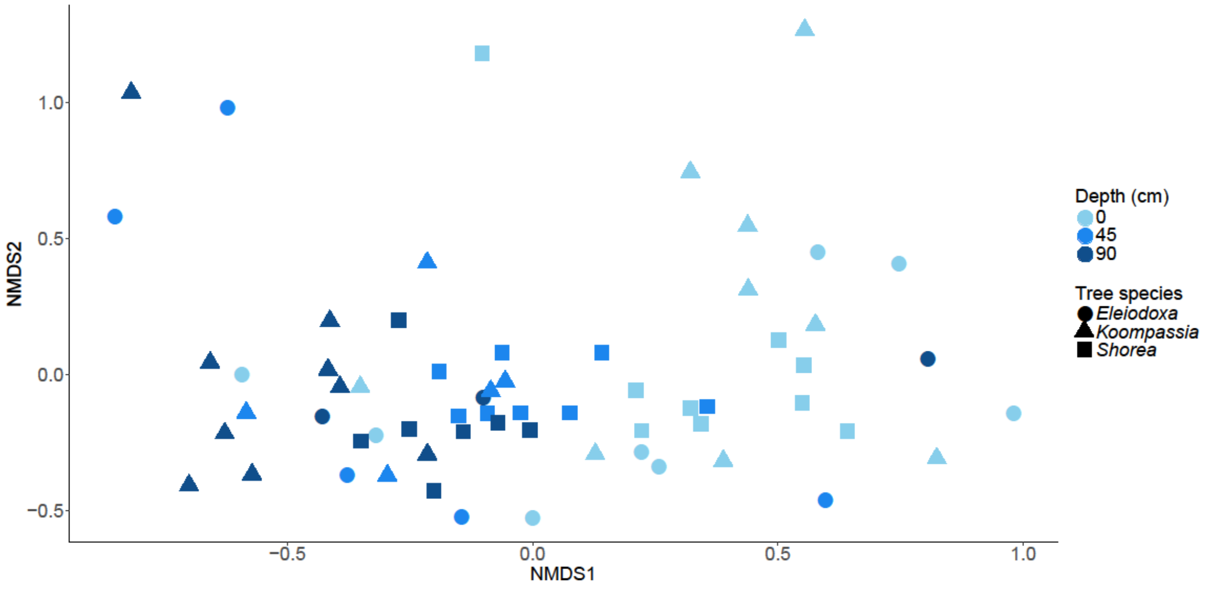

(b)

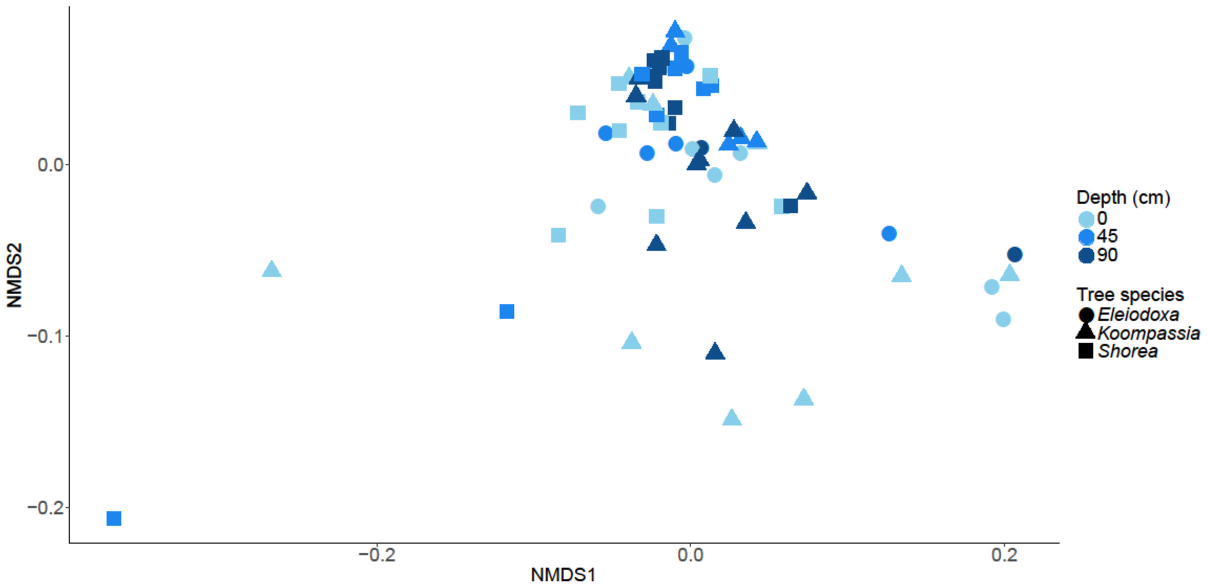

Figure S2. NMDS plot based on (a) Bray-Curtis dissimilarity and (b) weighted *UniFrac* distance of the archaeal communities in NSPSF.
